# MEMS Inertial Sensor Calibration Technology: Current Status and Future Trends

**DOI:** 10.3390/mi13060879

**Published:** 2022-05-31

**Authors:** Xu Ru, Nian Gu, Hang Shang, Heng Zhang

**Affiliations:** School of Computer and Information Science, Southwest University, Chongqing 400700, China; rxswu3214@email.swu.edu.cn (X.R.); gunianian@email.swu.edu.cn (N.G.); swushanghang@email.swu.edu.cn (H.S.)

**Keywords:** microelectromechanical systems, inertial sensors, accelerometer, gyroscope, background calibration, sensor fusion, error modeling and calibration

## Abstract

A review of various calibration techniques of MEMS inertial sensors is presented in this paper. MEMS inertial sensors are subject to various sources of error, so it is essential to correct these errors through calibration techniques to improve the accuracy and reliability of these sensors. In this paper, we first briefly describe the main characteristics of MEMS inertial sensors and then discuss some common error sources and the establishment of error models. A systematic review of calibration methods for inertial sensors, including gyroscopes and accelerometers, is conducted. We summarize the calibration schemes into two general categories: autonomous and nonautonomous calibration. A comprehensive overview of the latest progress made in MEMS inertial sensor calibration technology is presented, and the current state of the art and development prospects of MEMS inertial sensor calibration are analyzed with the aim of providing a reference for the future development of calibration technology.

## 1. Introduction

As a crucial part of MEMS technology, inertial sensors have been widely used to provide accurate position and motion measurement solutions [1] in many core areas such as aerospace, underwater exploration, robotics, healthcare, and portable devices (such as smartphones, computers, cameras, wearables, pedometers, etc.) [2,3,4,5,6,7,8,9]. MEMS inertial sensors, used for measuring angular velocity and acceleration [10,11], principally consist of micro-sensors, signal processing circuits, and microprocessors [12]. The most basic inertial sensors include accelerometers and gyroscopes (angular velocity gauges). An accelerometer is a sensor sensitive to linear acceleration and turned into a useable output signal; a gyroscope is a sensor that can be sensitive to the angular velocity of the motion of the moving body relative to the inertial space. These are the core components of inertial systems and are the main factors affecting the performance of inertial systems.

**Figure 1:**
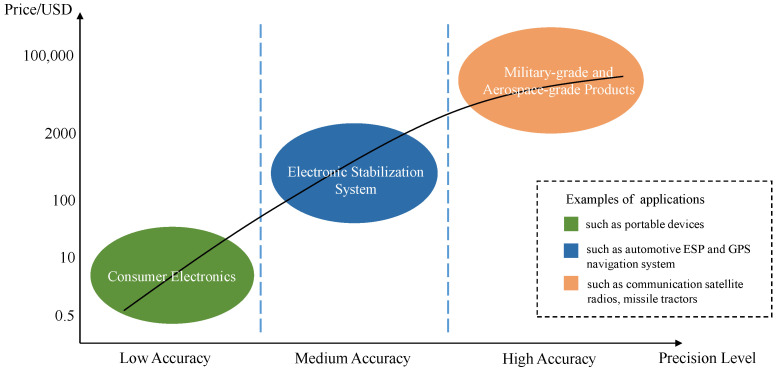
The cost of MEMS inertial sensors with varying degrees of accuracy in various application scenarios.

With the rise of frontier technologies such as navigation and positioning, autonomous driving, and personal wearable devices [13,14,15,16,17], the demand for inertial sensor accuracy is gradually increasing. In contrast, high-precision inertial sensors for high-end manufacturing (e.g., defense industry) are still difficult and costly to manufacture. The measurement results of MEMS inertial sensors will directly affect the output results of the carrier attitude in the navigation system, especially the gyroscope, whose drift affects the position error growth of the inertial guidance system as a cubic function of time [18], so the measurement accuracy of the sensor also directly affects the accuracy of the carrier attitude measurement in the whole navigation system. Precision measurement has become an inevitable issue in the development path of MEMS inertial sensors, while reducing their cost is also a current goal to pursue. Figure 1 shows the cost price of MEMS inertial sensors with varying degrees of accuracy in various application scenarios. Table 1 and Table 2 list the key parameters of sensors for different application levels.

MEMS inertial sensor errors are mainly related to the following factors: the misalignment and nonorthogonality of the sensitivity axes; the thermal and long-term drifts of the output signals (affecting both the bias and the scale factor); errors related to the installation and manufacture of sensors and aging of the silicon structure; variable bias due to thermal effects; and some uncertain noise sources or random errors [19,20,21,22]. Given these facts, this paper focuses on existing calibration techniques for MEMS inertial sensors and briefly analyses them. Calibration is the process of comparing the instrument output with known reference information and determining the coefficient that forces the output to agree with the reference information over a range of output values [23]. At the same time, certain calibration and test standards must be followed [24]. Using the method of error calibration, deterministic errors, such as offset, scale factor, nonorthogonality, misalignment and so forth, associated with the unknown orientation of the accelerometer and gyroscope sensitivity axes, thermal and long-term drifts of the output signals, installation and manufacture of sensors, aging of the silicon structure and so on can be effectively eliminated or compensated for [22]. This allows the sensor operating parameters to be accurately determined under given operating conditions, thereby increasing the accuracy and reliability of the sensor. For some random noise or error caused by random fluctuation of system response, it cannot be predicted or compensated directly owing to the drift in system response with time, which can only be characterized by a specific type of distribution and its statistical parameters and approximated by stochastic modeling [25,26].

The purpose of this paper is to systematically review different MEMS inertial sensor calibration schemes and their optimization methods, and to limit the discussion to IMU (accelerometer and gyroscope) calibration schemes in order to guide users in selecting the most suitable MEMS inertial sensor calibration method to improve sensor accuracy. The rest of the paper is organized as follows: In Section 2, we first discuss the error sources and error models of MEMS inertial sensors. Section 3 presents different calibration schemes for gyroscopes and accelerometers, and discusses them in two main categories. In Section 4, we discuss the current status of calibration techniques and the latest trends and present some observations on the current research. Finally, in Section 5, we propose the direction for the development of calibration techniques for MEMS inertial sensors.

## 2. Error Source and Error Model

**Figure 2:**
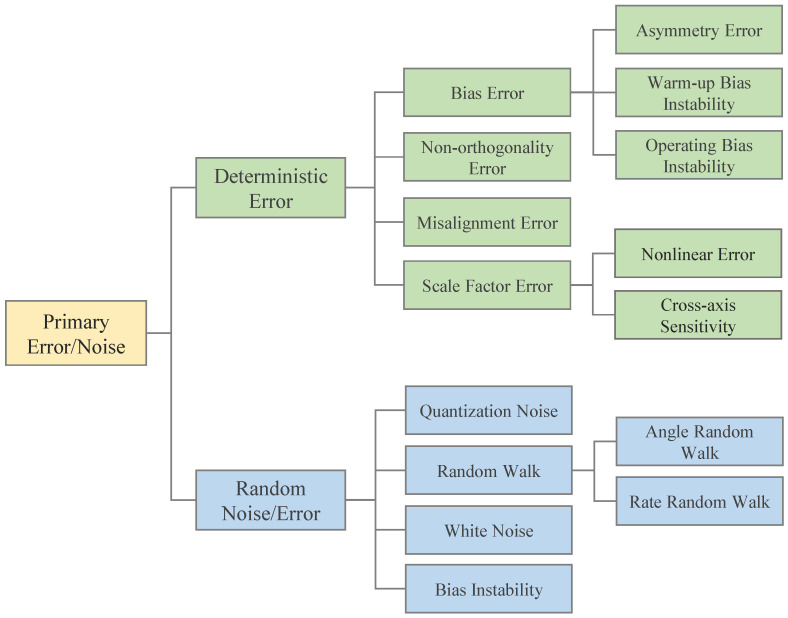
Some momentous errors and noise classification of inertial sensors.

As with any physical sensor, accelerometers and gyroscopes are subject to measurement errors. However, for these sensors, the error sources are multifaceted and coupled, which makes sensor calibration an important task [27]. The errors in accelerometers and gyroscopes can be roughly divided into deterministic and random errors [28]. Random errors include turn-on errors, random noise, and drift due to long-term operation, etc. [29,30], which are difficult to compensate for. Deterministic errors are caused by inaccurate factory calibration (or incorrect conversion parameters in the accelerometer datasheet) and include scale factor errors, bias, nonorthogonality errors, temperature-dependent data drift errors and so on. [29,30]. Among the deterministic errors that affect system performance, the most crucial ones are bias, scale factor, nonorthogonal and misalignment errors. As researchers continue to study mathematical models and algorithms in depth, errors caused by random noise have been greatly reduced. Due to the small size of MEMS, its accuracy is much less than some bulkier sensors. Therefore, deterministic errors have a greater impact on MEMS inertial sensors that are inherently less accurate. From the perspective of hardware structure, the micromechanical structure of inertial sensors is mostly made of silicon. Silicon is a temperature-sensitive material, and its physical properties vary greatly with temperature, which is also an important factor causing the thermal drift of offset and the scale factor, among which the thermal drift of offset is much more dominant [20,31]. This can be handled through the application of a temperature sensor and using the average thermal characteristics (or look-up table) of a specific accelerometer model [31]. Compared with thermal drift, the long-term drift and aging effect of a silicon structure are more difficult to compensate for, and some manufacturers choose to implement models to deal with the aging effect [19]. For long-term drift, you can try to repeat the calibration for a reasonable solution. It should be pointed out that the scale factor errors mentioned above are often also related to aging [32]. In addition, some errors caused by external environment changes (such as altitude and local and temporal changes of gravity acceleration) can also be compensated for by recalibration of the sensor. Figure 2 lists some important errors and noise of MEMS inertial sensor.

In order to more intuitively describe the error characteristics of inertial sensors by mathematical modeling, this paper assumes that in the three-dimensional motion space, in the carrier coordinate system, the actual measured physical vector of the three-axis accelerometer and gyroscope is expressed as $u={\left(u_{x},u_{y},u_{z}\right)}^{T}$, and its actual output is expressed as $v={\left(v_{x},v_{y},v_{z}\right)}^{T}$, where subscript *x*, *y* and *z* represent triaxial components. In addition, it should be added that the remaining error items will not be considered when discussing and analyzing the following errors separately in this section.

### 2.1. Deterministic Error

#### 2.1.1. Zero-Bias Error

**Figure 3:**
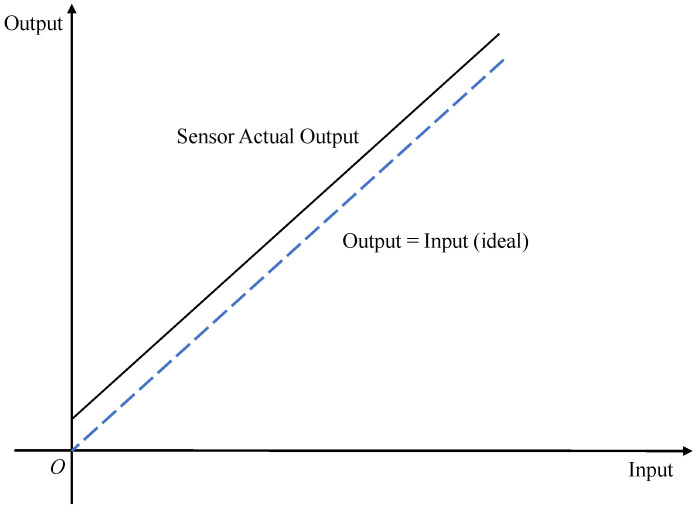
Zero-bias error schematic.

A zero-bias error, also known as zero-position error, can be subdivided into asymmetry error, warm-up zero-bias instability and operational zero-bias instability, and the measured values output from accelerometers and gyroscopes when the measured physical quantity is zero are usually called zero bias. As mentioned earlier, when the measured physical quantity is zero in the ideal situation, the output measurement data should also be zero. However, in the actual situation, the output is often not zero; there is a measurement error, and this error is called zero-bias error [33]. Figure 3 shows the schematic of zero-bias errors.

These errors are generally caused by a manufacturing error in the device. In addition, a zero-bias error is also one of the most important inertial sensor device errors, and for some devices, the zero-bias error is not fixed. In order to facilitate analysis, the zero-bias error can be divided into two parts, static and dynamic components, where the dynamic component is mainly affected by the change factors of the surrounding environment, such as temperature affecting the structure of tiny devices, etc. [34,35]. However, the percentage of dynamic component is tiny, about 10% of the static component, so generally at room temperature, the dynamic component is negligible. In addition, for the gyroscope, the g-dependent zero-bias error is also included in the zero-bias error of the gyroscope because the gravitational acceleration g also has some influence on it. Among the various types of inertial sensor devices, the zero-bias error exhibited by different devices is also different, and the zero-bias error of MEMS sensors is more pronounced [36]. Note that the zero bias of the triaxial accelerometer and gyroscope is $b={\left[b_{x},b_{y},b_{z}\right]}^{T}$. The zero-bias errors of both can be expressed as Equation (1).

(1) $$\left[\begin{array}{c} v_{x}\\ v_{y}\\ v_{z} \end{array}\right]=\left[\begin{array}{c} u_{x}\\ u_{y}\\ u_{z} \end{array}\right]+\left[\begin{array}{c} b_{x}\\ b_{y}\\ b_{z} \end{array}\right]=u+b$$

#### 2.1.2. Scale Factor Error

**Figure 4:**
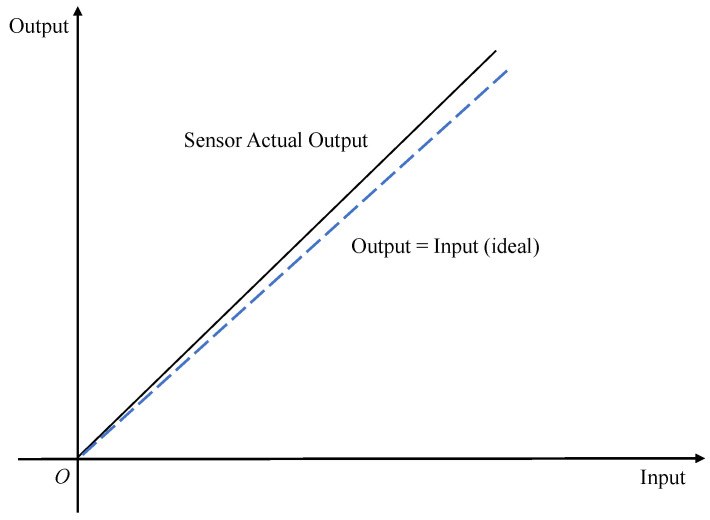
Scale factor error schematic.

The proportionality factor error is also called the scale factor error or sensitivity error. From a mathematical point of view, the scale factor generally refers to the ratio between the change in the output quantity and the change in the input quantity [37]. Then, for inertial sensor devices, the proportionality factor means that when the actual physical quantity measured by each axis changes, the output quantity that each axis is sensitive to will also change, and the ratio between the two is called the scale factor and has one dimension. Ideally, the scale factors of each axis should be the same (the dimension conversion ratio of the measurement result is not considered at this time); that is, the input change is the same as the output change. However, in practice, the scale factor is not only not one but also different; there is a measurement error, which is called the scale factor error. Figure 4 shows the schematic of the scale factor error.

Inertial sensors in the measurement process of each axis are measured separately, and each axis has a signal amplification circuit and a signal conversion calculation circuit for the measurement output. Due to manufacturing errors in the device, the characteristics of the circuit on each axis are not identical, resulting in a different sensitivity for each axis, which is the main reason for the proportionality factor error of inertial sensors. In addition, the scale factor error for a particular device is not definite because the structure of each measurement axis and the related circuit characteristics are susceptible to temperature, vibration, and other external environmental factors, so the corresponding scale factor error will also change accordingly. Under the general operating conditions at room temperature, the scale factor that can be considered to have an error is a constant. Note that the scale factor of the triaxial accelerometer and gyroscope are $s={\left(s_{x},s_{y},s_{z}\right)}^{T}$, and the scale factor errors of the two can be described by the diagonal matrices *S*, as shown in Equation (2). In the case that the scale factor error is 0, the scale factors both take the value of one.

(2) $$\left[\begin{array}{c} v_{x}\\ v_{y}\\ v_{z} \end{array}\right]=\left[\begin{array}{ccc} s_{x} & 0 & 0\\ 0 & s_{y} & 0\\ 0 & 0 & s_{z} \end{array}\right]\left[\begin{array}{c} u_{x}\\ u_{y}\\ u_{z} \end{array}\right]=Su$$

**Figure 5:**
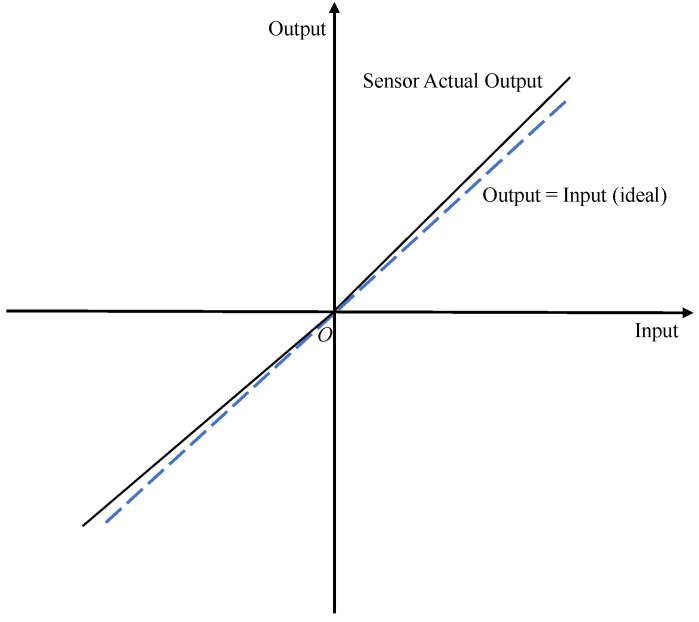
Scale factor asymmetry error schematic.

**Figure 6:**
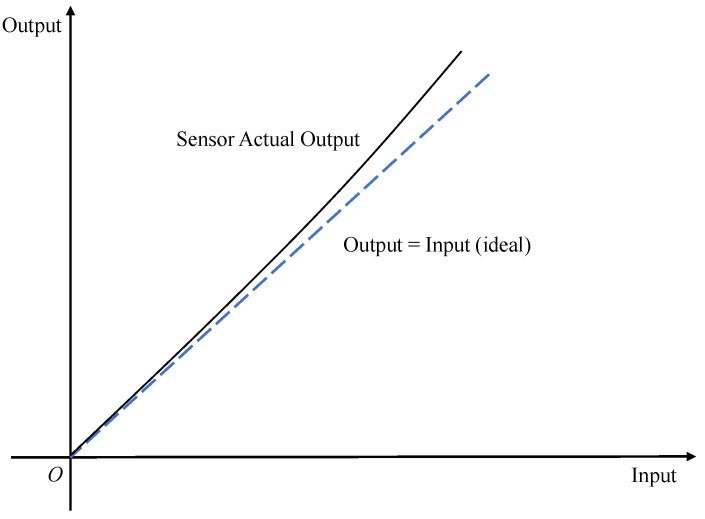
Scale factor nonlinear error schematic.

In addition, MEMS inertial sensors exhibit asymmetric and nonlinear properties in terms of scale factor error due to their lower cost and measurement accuracy. Asymmetry refers to the difference in scale factor error when the sensor measures the same physical quantity along the sensitive axis in the forward and reverse directions. Figure 5 shows a diagram of the asymmetry error of the scale factor. Nonlinearity refers to the variation of the scale factor with the magnitude of the sensor input [38]. Figure 6 shows a diagram of the nonlinear error of the scale factor. In general, a higher-order polynomial is used to represent the proportionality factor nonlinearity error.

#### 2.1.3. Cross-Axis Sensitivity

**Figure 7:**
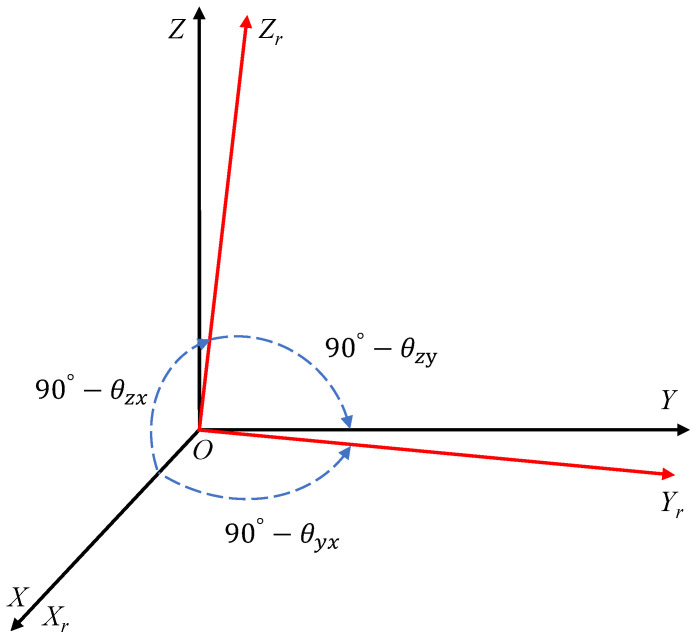
Nonorthogonality schematic.

The cross-axis effect is related to nonorthogonality and the crosstalk effect between different channels caused by the sensor electronics [39,40]. Among them, nonorthogonality error is the error that results from the procedure of imperfectly assembling individual sensors on an IMU chip or the inherent nonorthogonality caused by the fact that the sensitive axis in the sensor cannot be assembled perfectly due to the actual technical limitations in manufacturing [41,42]. This phenomenon can be explained by using Figure 7; the real axes of sensor is represented by using the nonorthogonal frame $X_{r}Y_{r}Z_{r}$, while the sensor frame O—XYZ is defined as an ideal orthogonal coordinate frame, and its *X*-axis is coincident with the axis $X_{r}$. The small angular errors $\theta_{yx}$, $\theta_{zx}$, $\theta_{zy}$ have to be estimated so that the nonorthogonal triaxial measurements can correctly project the orthogonal output readings with respect to the sensor frame [43]. Figure 7 shows the nonorthogonality schematic.

The cross-axis sensitivity of the triaxial accelerometer and gyroscope can be described by the third-order matrix *C*, as shown in Equation (3).

(3) $$\left[\begin{array}{c} v_{x}\\ v_{y}\\ v_{z} \end{array}\right]=\left[\begin{array}{ccc} 1 & c_{xy} & c_{xz}\\ c_{yx} & 1 & c_{yz}\\ c_{zx} & c_{zy} & 1 \end{array}\right]\left[\begin{array}{c} u_{x}\\ u_{y}\\ u_{z} \end{array}\right]=Cu$$

Here, it should be added that the element *c* in the matrix *C* is called the cross-axis factors, and there is no specific form for this matrix.

#### 2.1.4. Misalignment Error

**Figure 8:**
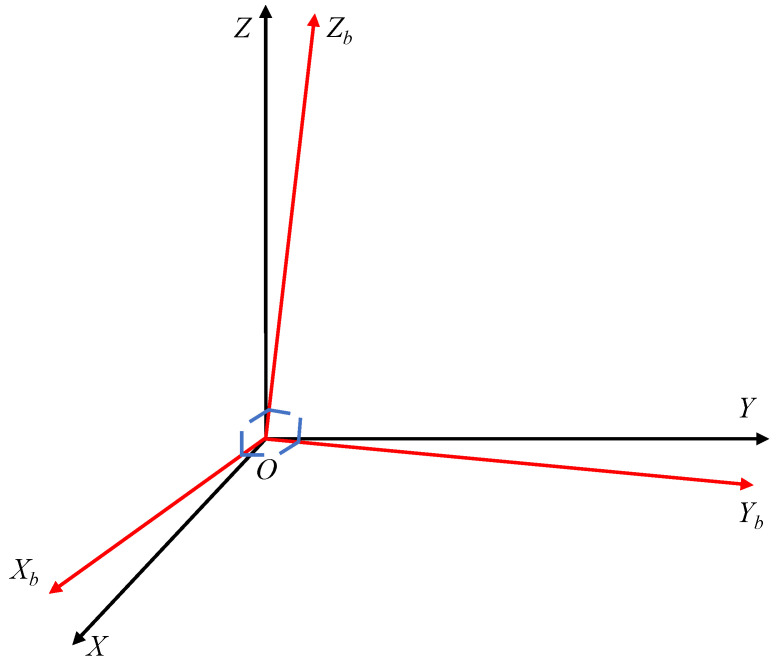
Misalignment schematic.

When the inertial sensor is installed on the carrier in the way of Jetlink, in theory, the three orthogonal sensitive axes of the sensor should be aligned with the three orthogonal axes of the carrier coordinate system of its carrier; i.e., the two axes should be aligned with each other. However, in practice, this is also due to the device processing and installation process errors, resulting in the installation of the sensor not being able to guarantee that the sensitive axis of the sensor and the carrier coordinate system of the coordinate axis coincide with the alignment fully, and so there is a mounting error angle between the two, which leads to measurement errors in the sensor, called misalignment errors [44]. Figure 8 shows the misalignment schematic.

The misalignment error of the triaxial accelerometer and gyroscope can be described by the third-order matrix *M*, as shown in Equation (4).

(4) $$\left[\begin{array}{c} v_{x}\\ v_{y}\\ v_{z} \end{array}\right]=\left[\begin{array}{ccc} m_{xx} & m_{xy} & m_{xz}\\ m_{yx} & m_{yy} & m_{yz}\\ m_{zx} & m_{zy} & m_{zz} \end{array}\right]\left[\begin{array}{c} u_{x}\\ u_{y}\\ u_{z} \end{array}\right]=Mu$$

Here, it needs to be added that, with the same generality of the form of the matrix *C*, the matrix *M* is also a general form for the description of misalignment errors, and this matrix also has no specific form. In fact, when only the misalignment error is considered, if the installation error angle of the sensor is determined, the specific forms of the matrix *M* can be obtained according to the coordinate transformation matrix formula; after inverse transformation of this, the misalignment errors can be calibrated. In addition, the elements in the matrices describing nonorthogonal and misaligned errors have no units and one dimension.

### 2.2. Random Error

Random errors are the errors that occur due to random variations of bias or scale factor drift over time and random sensor noise [34]. Stochastic variations in bias and scale factor are the low-frequency components of the random errors. The sensor noise is also a high-frequency component of the random errors [45]. The most important feature of random errors is that there may not be any direct relationship between input and output [46]. Allan variance tests and autocorrelation analysis are performed to determine the stochastic characterization of inertial sensors [47]. In addition to this, several stochastic processes exist for modeling random errors [45]. In this paper, the random errors are only briefly described, and the main stochastic processes associated with MEMS inertial sensor random error and noise are listed: [48]:

- Quantization noise (QN): When encoding an analog signal in digital form, a small amount of error may be introduced into the analog signal, which can be described as quantization noise [49];
- White noise (WN): This is defined as random unwanted values added to the real signal and differentiated by a long-term zero average [50]. Generally, the components of the noise spectrum of accelerometers and gyroscopes with frequencies below 1Hz are approximately regarded as white noise. In addition, white noise can neither be calibrated nor compensated for;
- Bias instability (BI): BI is one of the most important performance indicators of MEMS gyroscopes and is also considered as bias stability in some areas. BI is mainly calculated from the average standard deviation of the gyroscope output over a specific time frame, and it shows how the deviation changes with time at a constant temperature [51];
- Random walk (RW): RW includes angle random walk (ARW) and rate random walk (RRW). The angular random walk is caused by the random noise integration of the angular rate, which will eventually lead to random walk errors in the measured pose [52]. The rate random walk is caused by the random noise integral of the acceleration, which can cause random walk errors in the inertial navigation velocity calculation. The errors caused by both have the characteristics of random walk.

### 2.3. Error Model

Before establishing the error model of MEMS inertial sensors, we first make the following assumptions based on the analysis of each error characteristic of the sensors, together with some experimental conditions:

1. The influence of the external temperature and magnetic field environment on the sensor error is ignored; that is, it is assumed that in the current time and space, the measured vector field and the deterministic error of the sensor are time invariant;
2. For the convenience of discussion, it is assumed that the higher-order term of the systematic error of the sensor is negligible; that is, the nonlinear part of the systematic error is not considered, and only the linear error is considered;
3. For the random noise error of the sensor, because for the general random noise the mean value of the random error tends to 0 when the amount of data reaches a certain level, this paper assumes that the random error of the sensor is Gaussian noise with a mean value of 0, we mainly calibrated the deterministic error of the MEMS inertial sensor.

Combined with the analysis of various error characteristics of the aforementioned accelerometer and gyroscope, based on the above assumptions, the error model of the three-axis strapdown accelerometer and gyroscope are established as Equation (5):

(5) $$\begin{array}{cc} \left[\begin{array}{c} v_{x}\\ v_{y}\\ v_{z} \end{array}\right] & =\left[\begin{array}{ccc} m_{xx} & m_{xy} & m_{xz}\\ m_{yx} & m_{yy} & m_{yz}\\ m_{zz} & m_{zy} & m_{zz} \end{array}\right]\left[\begin{array}{ccc} 1 & c_{xy} & c_{xz}\\ c_{yx} & 1 & c_{yz}\\ c_{zx} & c_{zy} & 1 \end{array}\right]\left[\begin{array}{ccc} s_{x} & 0 & 0\\ 0 & s_{y} & 0\\ 0 & 0 & s_{z} \end{array}\right]\left[\begin{array}{c} u_{x}\\ u_{y}\\ u_{z} \end{array}\right]+\left[\begin{array}{c} b_{x}\\ b_{y}\\ b_{z} \end{array}\right]=MCSu+b \end{array}$$

where *u*, *v*, *b*, *S*, *C* and *M* are the input, output, bias error, scale factor error matrix, cross-axis sensitivity matrix and misalignment error matrix of the accelerometer and gyroscope, respectively.

Since each error matrix in the above formula is a general description of the corresponding error, not a specific form, in order to simplify the discussion, this paper simplifies Equation (5) to Equation (6):

(6) $$\left[\begin{array}{c} v_{x}\\ v_{y}\\ v_{z} \end{array}\right]=\left[\begin{array}{ccc} k_{xx} & k_{xy} & k_{xz}\\ k_{yx} & k_{yy} & k_{yz}\\ k_{zx} & k_{zy} & k_{zz} \end{array}\right]\left[\begin{array}{c} u_{x}\\ u_{y}\\ u_{z} \end{array}\right]+\left[\begin{array}{c} b_{x}\\ b_{y}\\ b_{z} \end{array}\right]=Ku+b$$

where *b* is the constant term error of the accelerometer and gyroscope and *K* is the combined error matrix of the accelerometer and gyroscope. In addition, according to the aforementioned assumptions in this paper, each element in the constant value term error *b* and the proportional term error matrix *K* are undetermined constants, that is, unknown time-invariant coefficients. The error model described by Equation (6) is a linear expression.

To sum up, the error model established by the mathematical modeling method for the MEMS inertial sensor is consistent from a mathematical point of view, and can be unified into the form of Equation (7).

(7) $$v=\left[\begin{array}{c} v_{1}\\ v_{2}\\ v_{3} \end{array}\right]=\left[\begin{array}{ccc} k_{11} & k_{12} & k_{13}\\ k_{21} & k_{22} & k_{23}\\ k_{31} & k_{32} & k_{33} \end{array}\right]\left[\begin{array}{c} u_{1}\\ u_{2}\\ u_{3} \end{array}\right]+\left[\begin{array}{c} b_{1}\\ b_{2}\\ b_{3} \end{array}\right]=Ku+b$$

where *u* is the input of the sensor, *v* is the output of the sensor, *b* is the constant term error of the sensor, and *K* is the proportional term error matrix of the sensor. For different sensors, although the form of the error model is the same, the error sources are different and the unknown error parameters are not the same. The idea of the mathematical modeling approach is to not consider the specific physical mechanism and error sources, but only the mathematical relationship between the input and output. In practical applications, in addition to the known error terms that have been analyzed above, other errors proportional to the input vector contained in the error can also be described as a whole by *K*, and the error of the constant term can be described by *b* as a whole. Therefore, the error model has its rationality and practicability from both a mathematical point of view and the practical application point of view.

After the error model of the sensor is determined, the unknown error parameters in the error model need to be determined by certain mathematical methods. In the aforementioned process of the error calibration of the sensor, the error calibration of the sensor is essentially to first calibrate the unknown error parameters *K* and *b* in the error model v=Ku+b, and then according to $u=K^{-1}\left(v-b\right)$ calculate the exact value of the measured physical quantity from the actual output value of the sensor, thereby realizing error compensation. Ref. [53] illustrates the detailed characterization and calibration process of a MEMS-based IMU by employing the 3DM-GX1 MEMS inertial sensor, and gives an overview of the estimation of each error calibration parameter.

## 3. Calibration Methods

Calibration is used to solve the error coefficient by comparing the difference between the actual measured output of the sensor and the preset reference value, and constructing the relationship between the measured output and the expected output, so that the measured output of the sensor tends to be consistent with the ideal output. Through the difference of preset reference value or reference object, it can be divided into two categories—autonomous calibration and nonautonomous calibration. The specific difference lies in whether it needs to be assisted by high-precision equipment in the laboratory, that is, through the attitude reference datum provided by large precision instruments to perform error calibration.

- Nonautonomous calibration: this calibration needs to be assisted by precision instruments with good structure, such as a high-precision turntable, centrifuge, shaking table, and so on [54,55,56].
- Autonomous calibration: without the assistance of high-precision instruments, this calibration is completed by using the external reference excitation provided by the local gravity field, the rotational angular velocity of the earth, uniform magnetic field, and so on.

The demand for improving the calibration accuracy of the sensor is increasing. At the same time, it has been proved by experiments that since the calibration parameters are given by the parameter estimation algorithm, the use of an appropriate parameter estimation algorithm can effectively improve the calibration accuracy [57]. Therefore, before discussing the relevant calibration methods in detail, we first introduce the parameter estimation algorithm closely related to calibration. When enough effective data samples are collected, we need to determine or estimate the error parameters in the error model through appropriate mathematical methods. A more accurate, faster convergence, lower complexity and more stable parameter estimation algorithm is our common pursuit.

### 3.1. Parameter Estimation Algorithm

(1) Least Squares

In essence, the least squares (LS) algorithm is used to explore the minimum error between the observed quantity and the target quantity. Here, we can understand it as the error between the measured data obtained by the accelerometer or gyroscope and the expected data. Considering the nonlinearity of the deterministic error model, we usually use the nonlinear least squares method to estimate the optimal parameters, such as the gradient descent method, Newton’s method [39,58], Gauss–Newton iteration method (GN) [59], Levenberg–Marquardt method (LM) [60], etc. On the other hand, the nonlinear problem can be transformed into a linear problem through some skills. The linear least square method can be used to solve it, such as normal equation, Cholesky decomposition, singular value decomposition, etc.

(2) Maximum Likelihood Estimation

The maximum likelihood estimation deals with problems from the perspective of probability, but usually uses the probability density function instead of probability. It can be seen that the maximum likelihood estimation requires a certain understanding of the global probability density. Here, assuming that the noisy model conforms to the Gaussian process, and the probability density function also obeys the Gaussian distribution, the maximum likelihood estimation can be used to solve it [61].

(3) Kalman Filtering and its extensions

Kalman filtering and its extensions attempt to estimate the carrier state and calibration parameters simultaneously in a framework according to the system model and observations over a period of time [62]. Here, continuous rotation excitation can be performed through the turntable, or other conditions, such as acceleration, electrostatic force, etc., can be taken as the observed quantities.

(4) Other Intelligent Optimization Algorithms

The particle swarm optimization algorithm is a random search method based on group cooperation. It realizes the update of speed and position by tracking two extreme values, with memory function and a high convergence speed [63]. In addition, the neural networks can calibrate the output without explicitly deriving the error model and estimating the nonlinear parameters [64].

Although most of the existing algorithms can accurately identify the unknown parameters, they have strict requirements for the initial value, cannot always ensure convergence, and are relatively complex to embed in a microcontroller unit [65]. Therefore, the improvement of calibration accuracy can not only start from model optimization and the reference standard selection, but also from the optimization of parameter estimation algorithms. Next, through the cross-combination of parameter estimation algorithms and calibration schemes, the calibration methods of accelerometer and gyroscope will be discussed, addressing two types: nonautonomous calibration and autonomous calibration.

### 3.2. Accelerometer Calibration Method

**Figure 9:**
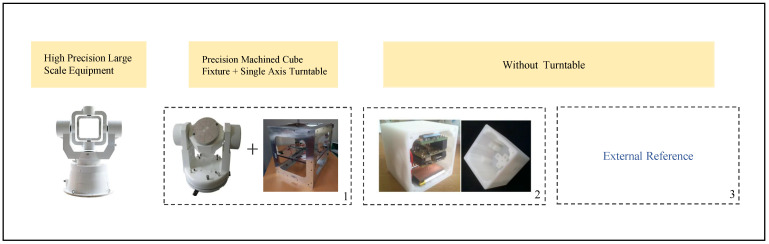
Some crucial existing calibration methods. Box 1 represents the combination of a low-precision turntable and cube fixture [66]; box 2 represents the tilt sensor with 3D-printed housing [22]; box 3 represents other autonomous calibration methods that do not require a turntable.

The traditional laboratory method of the accelerometer is to provide an attitude reference through precision instruments such as a three-axis turntable. In order to reduce the dependence on the turntable, the precision-machined cube housing can be introduced as an aid so that the strong requirement for the accurate value of gravitational acceleration can be avoided by selecting a specific attitude. In order to better realize the on-site calibration, add the constant vector of gravitational acceleration or other external excitation to constrain, which not only gets rid of the limitations of high-precision instruments in the laboratory, but also does not require the accurate value of local gravitational acceleration, so as to broaden the calibration environment further. The acceleration calibration is shown in Figure 9.

#### 3.2.1. Nonautonomous Calibration

Traditional nonautonomous calibration usually uses two-axis or three-axis turntables [35,67]. The purpose of these high-precision large-scale instruments is to make the inclination angle of the sensor accurate, knowable, and controllable. In the laboratory environment, the accelerometer to be calibrated is placed horizontally in a piece of laboratory equipment, such as a turntable, and the multi-position rotation calibration based on gravity is adopted to solve the unknown parameters in the error model according to the theoretical acceleration (reference value) and measured value of the determined position and angle.

The most classic algorithm for nonautonomous calibration is the static six-position calibration method. Keep the accelerometer system coordinate system consistent with the turntable test coordinate system, and test each sensitive axis of acceleration vertically up and down with the help of high-precision turntable. Once each sensitive axis senses the acceleration of *g* and then −g, the bias *b* (Equation (8)) and the scale factor *s* (Equation (9)) are calculated [22].

(8) $$b=\frac{f_{up}+f_{\mathrm{down}\;}}{2}$$

(9) $$s=\frac{f_{up}-f_{\mathrm{down}\;}}{2K}$$

where $f_{up}$ is the measured value of the sensor when the particular sensitive axis is facing up, $f_{\mathrm{down}\;}$ is the measured value when the sensitive axis is facing down, and *K* is the known reference signal. For accelerometers, *K* is the local gravity constant *g* [28].

In static six-position calibrations, the sensitive axis of the sensor is aligned with the vertical axis of the local-level frame by default, ignoring nonlinear errors, and the crosstalk effects caused by the nonorthogonality error between axes [68]. The accuracy largely depends on the degree of alignment. On the one hand, all sensors can be calibrated to the same frame simultaneously by adding a cube housing to reduce the misalignments between the measurement plane and the sensor. On the other hand, more unknown error parameters can be estimated using a multi-position calibration by optimizing the error model.

Ref. [69] proposed a tilt-compensated digital compass accelerometer output calibration method assisted by precision aluminum cube housing, which realizes error calibration by providing six mutually orthogonal directions, east, south, west, north, sky and earth—twelve positions. However, the introduction of precision cube housing does not mean turntable is not required. It is only used to reduce the misalignment error between the turntable and the sensor. The algorithm cannot work when the tilt angle is ±90, which further increases the limitation of the algorithm.

For multi-position calibrations, Ref. [70] pointed out two key problems:

- Inter-triad misalignment calibration between gyroscope and accelerometer;
- Optimal design of the calibration scheme.

Moreover, through fixed axial rotation, the rotation axis of the accelerometer is used as the reference of the rotation axis of the gyroscope to solve the inter-triad misalignment problem between the gyroscope and accelerometer; by maximizing the sensitivity of the norm to the calibration parameters, an optimal accelerometer calibration scheme with nine attitudes is obtained. Table 3 is the schematic diagram of the optimal scheme for accelerometer calibration with nine attitudes. However, in the process of calibration, these coefficients will change slowly with time, which reduces the efficiency of offline calibration. If all the error parameters in the accelerometer model are estimated online, it is evident that the unknown coefficients are too large to be realized.

Ref. [71] simplified the model by conducting online calibration only for bias, which varies dramatically over time and electricity. First, the off-line calibration eliminates the scale factor and nonorthogonality error, and then the on-line calibration of the dynamic bias is based on the time-varying Kalman filter. The effect of the lever arm on accelerometer calibration is not taken into account.

In order to further improve the accuracy of the sensor and reduce the impact of the lever arm effect, Ref. [72] proposed a prediction error minimization stochastic modeling suitable for the skew redundant inertial measurement unit, which takes into account not only the static errors—bias, scale factor, and misalignments—but also the dynamic errors caused by gyroscope angle random walk and accelerometer velocity random walk. Compared with the traditional least squares method [28,73,74], which can lead to a nonoptimal estimation and nonactive bias, a dual Kalman filter estimation method is proposed, which simulates the influence of bias instability and random walk noise on the Kalman filter, reduces the bias estimation, and completes the parameter convergence in dynamic motion. Similarly, Ref. [75] introduced an angular acceleration estimator to improve the additional error produced by the calibration of the turntable. He proposed a nonlinear model, considering that the output of the accelerometer is affected by the arm effect, installation error and the distance from the center of the turntable, and estimated the model parameters through the use of a transformed unscented Kalman filter (TUKF). However, due to the necessity of introducing an angular acceleration estimator, this calibration is limited to laboratory environments.

Although nonautonomous calibrations require expensive laboratory equipment and a high calibration time, its high calibration accuracy makes it still the first choice for many large companies and enterprises.

#### 3.2.2. Autonomous Calibration

The traditional split calibration above relies on large-scale turntable equipment, and the calibration accuracy depends more on turntable equipment. The use environment is limited, the operation is cumbersome, and data acquisition takes a long time. The on-site calibration technology of abandoning the turntable—autonomous calibration—came into being.

The basis of accelerometer autonomous calibrations is that the gravitational acceleration is a constant vector (Equation (9)), so we can no longer rely on the turntable, but start from the characteristics of the accelerometer; that is, in the static state, the two-norm acceleration measurement value is always equal to the local gravitational acceleration (Lötters et al. [77]), with modulo-length invariance in the free-rotation case, thus constraining the acceleration. It should be pointed out that the gravitational acceleration as a reference is justified only in the case of low-range accelerometers.

(10) $${Acc_{x}}^{2}+{Acc_{y}}^{2}+{Acc_{z}}^{2}=G^{2}$$

Based on this original idea, we can explore how to transform the above nonautonomous calibration into autonomous calibration.

(1) Cube Housing

Ref. [78] certified that the use of suitable housing, preferably cube housing, is beneficial for calibration. Different from the above [69], Ref. [66] only used cube housing and a fixture to calibrate all sensors to the same frame at the same time. Equation (11) is used as calibration model and the high-precision cube frame is used as a rotating tool to change the sensor position, so as to minimize the residual of overdetermined equations and estimate the direction of gravity and more calibration parameters.

(11) $$\tilde{g}\left(t_{k}\right)=S_{a}C_{c}^{s}\left(t_{k}\right)C_{l}^{c}g+b_{a}+\epsilon_{a}$$

where $C_{l}^{c}$ maps the references from local frame, where the reference signals are presented, to the calibration frame, where the calibration rotations and matrix $C_{c}^{s}\left(t_{k}\right)$ map the reference from the calibration frame to the sensors’ frame. g is the original output, $\epsilon_{a}$ is noise, $b_{a}$ is bias, and $S_{a}$ is a nonsingular constant scale matrix. $\tilde{g}\left(t_{k}\right)$ is the mean of the gravity.

The use of specially designed cube housings can be easily and quickly recalibrated by the user in order to eliminate such considerable errors as thermal and long-term drifts as well as effects of aging, keeping the sensitive axes aligned with respect to the external mechanical datum [22].

(2) Multi-position

Ref. [77] first proposed a multi-position method based on ellipsoid fitting that does not require a turntable. The IMU output signal passes through the high-pass filter, the rectifier and the low-pass filter successively, and the output is Equation (12),

(12) $$v_{\mathrm{out},\mathrm{detection}\;}=\mathrm{LPF}\left(\mathrm{REC}\left(\mathrm{HPF}\left(v_{\mathrm{in},\mathrm{detection}\;}\right)\right)\right)$$

However, the nonorthogonality error is not considered and, in the nonstationary state, it is necessary to know the value of each moment and the computation is large.

Based on the original idea of accelerometer autonomous calibration [77], Ref. [58] derived a cost function equation, Equation (13), from the error model to describe the deviation between the square of the accelerometer measurement ${∥h\left(A_{k}^{s},X\right)∥}^{2}$ and the square of the gravitational acceleration ${∥g∥}^{2}$, and used the Newton iterative method to estimate the accelerometer parameters.

(13) $$G\left(X\right)=\sum_{K=1}^{L}{\left({∥h\left(A_{k}^{s},X\right)∥}^{2}-{∥g∥}^{2}\right)}^{2}$$

Its convergence speed is fast, but it has strict requirements for the initial value [79]. The LS method usually gives the nonoptimal estimation of calibration parameters and may lead to nonactive bias in the estimation. In particular, this method does not consider the influence of the bias instability of MEMS inertial sensor residuals in the calibration process. Therefore, Ref. [65] proposed a six-point G-optimal experimental scheme for the special six-parameter second-degree model based on DoE. A convergence-guaranteed recursive parameter estimation algorithm is developed since the nonlinear parameter estimation method cannot always converge ideally, and the algorithm’s efficiency is low. It can realize the accurate estimation of parameters in three iterations and the response of parameter estimation is stable for different initial values. Ref. [80] used the generalized nonlinear least square (GNLS) method to estimate the deterministic error. Compared with the GN iteration method and LM method, it is proved that the convergence speed of the scheme is better, but it takes more time to collect data from 30 locations.

(3) Other External Equipment

Ref. [81] proposed a calibration method of an accelerometer without physical stimulation, which realizes nonphysical excitation calibration by using the electrostatic force applied at transducer test plates to excite the proof mass. The purpose is to replace the physical acceleration with electrostatic force, and use the extended Kalman filter algorithm to estimate the gain parameters, which is helpful to evaluate the linearity and sensitivity of the equipment. Ref. [82] proposed a closed-loop self-calibration system for accelerometers, combining the sensor and actuator. In the self-calibration system, the standard acceleration environment provided by the microvibrator is realized through a closed-loop control based on the embedded optical displacement sensing system. Through the designed self-calibration process, the accelerometer’s error coefficient and compensation coefficient are obtained.

In addition, there is no robust method to calibrate the acceleration output without explicit derivation of the error model and estimation of the nonlinear error parameters. Ref. [64] presented a new calibration algorithm based on neural networks. Using the neural network with the back-propagation and optimization method, the actual measurement and the expected value of the accelerometer at rest are used as training data to find the best model to match the sensor’s output signal with the acceleration of static position.

Although the autonomous calibration of accelerometers involves extensive formula derivation, lengthy operations, and attention to the convergence of the estimation algorithm during specific runs, it can be used in-field calibration compared with the nonautonomous calibration, and does not require a high-accuracy operation. Through optimization, the accuracy is also gradually aligned to the nonautonomous calibration.

### 3.3. Gyroscope Calibration Method

Since the rotational angular velocity of the earth is a weak signal, that is, the earth rotation speed is hidden in large noise, only gyroscopes with medium or higher accuracy can sense it acutely, so the calibration of gyroscopes requires more external excitation than accelerometers, such as large-scale instruments—centrifuges, motion rate tables, etc.—and other external excitation—electrostatic force, gravitational acceleration, etc.

#### 3.3.1. Nonautonomous Calibration

Nonautonomous calibration is dependent on a mechanical platform, rotating the IMU into different, predefined, precisely controlled orientations and angular rates. The gyroscope can be calibrated theoretically through Equations (8) and (9), in which *K* represents the vertical projection of the earth’s rotation rate in a fixed dimension [28]. Ref. [83]’s experiment shows that due to the small amplitude of the earth’s rotation rate, it is unacceptable to calibrate the accuracy of the gyroscope using the static six-position method, and calibrating the gyroscope requires greater rotation excitation. Ref. [79] provides greater rotation excitation through a turntable with a precision of 0.1 °/s, noting that the accelerometer and the gyroscope are calibrated separately. Therefore, it is impossible to estimate the inter-triad misalignments between the gyroscopes and accelerometers.

Ref. [84] proposed an improved scheme based on multi-position calibration [83], which is optimized for the two shortcomings of gyro multi-position calibration.

- Inability to sense weak signals of the earth’s rotation.
- Stringent requirements for initial values.

This scheme does not need leveling, but ignores the difference between the angular velocity of the spindle and that of the reverse rotating platform. The accelerometer and gyroscope are independently calibrated, so the calibration accuracy depends on the degree of alignment.

In order to solve the problem that the gyro calibration is sensitive to the input angular rate and the angular rate is affected by the difference between the spindle rate and the reverse rotating platform rate, Ref. [85] further analyzed the possible error sources on the turntables or centrifuges and established the corresponding coordinate system. By combining the output of 16 specific positions, the angular velocity derived by the centrifuges, the input specific force and the static error model of the gyroscopes, the expression of the bias coefficient is obtained to improve error compensation precision.

In response to the independent calibration of acceleration and gyroscope, ignoring the inter-triad misalignments, Ref. [86] in 2008 took advantage of the fact that the dot product of accelerometer and gyroscope output is directly related to the dot product of the earth’s rotation rate and gravitational acceleration to increase the constraint between accelerometer and gyroscope. However, the accuracy of the error parameters of the gyroscope obtained by this method needs to be improved. Ref. [76] considered the combined calibration of accelerometer and gyroscope, and proposed a new method to calibrate the inter-triad misalignments between gyroscope and accelerometer, by constructing the following model Equation (14),

(14) $${\tilde{\omega }}_{ig}^{g}=K_{g}T_{o}^{g}R_{p}^{o}\omega_{ip}^{p}+b_{g}+v_{g}$$

where $\omega_{ip}^{p}$ denotes the true platform angular velocity with respect to the inertial coordinates expressed in platform coordinates, $K_{g}$ is the diagonal scale factor matrix, $b_{g}$ is the bias vector of the gyroscope cluster, $v_{g}$ is the measurement noise, $R_{p}^{o}$ denotes the directional cosine matrix, rotating a vector from the platform coordinates to the o-frame, and $T_{o}^{g}$ represents the nonorthogonality error between the orthogonal coordinate system o-frame and the sensitive axis of the gyroscope.

Ref. [87] introduced a thermal chamber. After estimating scale factors, misalignments and biases, thermal calibration was performed to determine the scale factors and biases at different temperatures, and a Kalman filter was used to derive the optimal calibration algorithm.

Although nonautonomous calibrations can have higher accuracy through the introduction of turntables, centrifuges and so on, many scholars have also noticed that the introduction of large-scale equipments will also bring new errors. And when the sensors are integrated in a frame, the mutual crosstalk between the two also needs to be considered. Therefore, it needs to be further improved through fine division of error sources and optimization of models.

#### 3.3.2. Autonomous Calibration

For the autonomous calibration of gyroscopes, in addition to the rotational angular velocity of the earths, some other auxiliary excitation are used—accelerometers, magnetometers, etc. The following mainly explains the autonomous calibration of the gyroscopes under different auxiliary excitations:

(1) Multi-position

**Figure 10:**
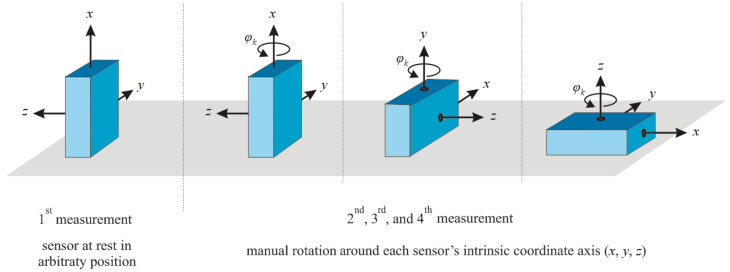
Orientations of the 3D gyroscope during the four calibration measurements [78].

Ref. [78] realized the estimation of 12 position parameters through the four measurements in Figure 10, and only required manual rotation to complete the gyroscope calibration. However, many assumptions are set, and the robustness and generality of its algorithm cannot be guaranteed.

(2) Accelerometer

Ref. [88] first calibrated the accelerometer through the six-position method, then determined the wheel speed with the help of the accelerometer, and proposed a scheme for calibrating the gyroscope by combining the accelerometer and the bicycle wheel, so the error caused by the accelerometer will be superimposed on the gyroscope. Ref. [89] calibrated the gyroscope by comparing the output of the accelerometer and the directional integral of the IMU after any motion. It is necessary to roughly estimate the gyroscope parameters. In addition, theoretically, the gravitational vector of the accelerometer should be equal to the calculated gravitational vector through the output of gyroscope, so some scholars adopted the form of combination of motion and static state, and use the error parameters under static state as the initial values under dynamic motion to improve the precision.

Refs. [58,90] eliminate the limitation of sensor orientation by introducing the gravitational field of the earth so that the calibration of the gyroscope can be achieved only by simple rotation. However, the gyroscope must be rotated in a fixed structure. Ref. [66] utilizes the known rotations between positions in a multi-position calibration to correlate the first and last quaternions of each rotation with the corresponding 24 reference positions, so that the calibration process does not require precise alignment, enabling the rotation plane freedom.

(3) Magnetometer

Ref. [91] proved that the uniform magnetic field vector could be used as a reference for low-cost gyroscopes under enough rotation excitation, and the gyroscopes can be calibrated in a uniform magnetic field by using a magnetometer. However, it requires high magnetic field stability, which cannot be used as a reference when the magnetic field is disturbed by an external alternating magnetic field. Ref. [92] put forward a complete solution to calibrate the inertial sensor, which can calibrate the inertial measurement unit in the case of dynamic magnetic interference.

(4) Electrostatic Force

Ref. [93] mimicked the effect of the Coriolis force by the application of a rotating excitation to the drive and sense resonance modes of the device. The purpose of this is to utilize the condition that the phase shift of the vibration mode is proportional to the excitation rotation rate so that the rotating excitation generated by the rotating electrostatic field applied to the equipment electrode replaces the physical rotation.

From the description of various calibration algorithms in Table 4, we can see that it is difficult to determine the merits of the algorithm from just one aspect, such as cost, accuracy, complexity, etc. It is more a choice based on specific scenarios. For example, the static six-position method and rate calibration, which need the assistance of precision instruments, are still the first option for calibration by most enterprises. However, it is worth considering the appropriate exchange of accuracy for operation efficiency and scene expansion in practical applications.

## 4. Discussion

### 4.1. Current Situation Analysis

Based on a large number of literature materials, we conducted an extensive comparative analysis of the existing research methods proposed for MEMS inertial sensor error calibration and summarized based on this. So far, many effective error calibration methods for MEMS inertial sensors have been proposed by researchers in this field for different practical applications and have achieved good results, as well as good practicality and applicability. As far as the autonomous calibration method is concerned, the classical independent ellipsoid fitting method and the independent dot product invariant method are proposed to guarantee the error calibration effect from the mathematical principle, and are easy to use. However, the application of this method to a single device calibration has its inherent drawbacks, such as the ellipsoidal fitting method which introduces artificial misalignment errors to the sensor during calibration, and the independent dot product invariant method which requires an internally predetermined reference vector, which is not available and difficult to implement in many practical applications. Moreover, there is no unified method for error calibration of multi-sensor combinations, especially in terms of autonomous calibration.

As for nonautonomous calibration, the input and output data sequences of the sensors are obtained by artificially creating vector fields under laboratory conditions, for example, by applying a centrifuge or a high-precision turntable to calibrate triaxial accelerometers [60,94]. This type of calibration method is not only expensive in terms of investment in equipment and experimental conditions, but also the calibration process can only be performed in a dedicated artificial laboratory environment, which is not universal to the actual application environment, so it is not suitable for low-cost application scenarios.

**Figure 11:**
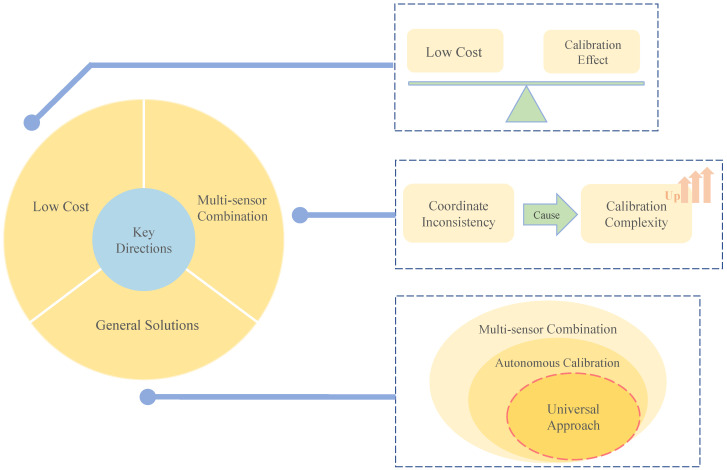
Schematic of key directions. On the right is a further exploration of three key directions, from top to bottom, the balance between low cost and calibration effect, the impact of coordinate inconsistency on multi-sensor combinations, and the lack of a universal solution for autonomous calibration of multi-sensor combinations.

Based on this, we identified three key directions to focus on for error calibration of MEMS inertial sensors, especially when considering multi-sensor combinations (Figure 11).

- In order to effectively take advantage of the low cost of the MEMS inertial sensor, it should be considered that the error calibration method it adopts should also meet the requirements of being low cost while achieving the calibration effect;
- For the error calibration of the multi-sensor combination, it is necessary to consider avoiding the problem of complicating the error calibration caused by the nonuniform coordinates of the multi-sensor combination;
- The current research does not propose a unified and general solution for error calibration of multi-sensor combinations, especially in terms of autonomous calibration.

In a word, because the method of improving the hardware of the sensor is not only tricky and the research cost is high, but also its related technology is also restricted by the current research level in the entire field. In practical operation, there will also be technical limitations in various links such as product processing, packaging, testing and equipping, which are not applicable in most application scenarios. Therefore, at present, improving the accuracy of MEMS sensors mainly adopts the software level method and develops towards low cost. Based on the existing device development level, the product performance is improved by the error calibration method.

### 4.2. Future Trends

As for the future direction of MEMS inertial sensor calibration technology, our view is towards diversification. On the one hand, combining cameras with IMUs to form vision-aided inertial navigation systems (VINS) has been a hot research topic [95]. The camera and IMU are complementary in terms of accuracy and frequency response. Specifically, the IMU can provide scale information and robustness for dynamic motion, while the camera can estimate the maximum scale pose. In the work of IMU-camera joint calibration, it can be traced back to Alves et al.’s proposal to install visual-inertial sensors on a pendulum and estimate the relative orientation between the camera and the IMU, as well as the scale and axis misalignment parameters of the IMU [96]. Refs. [97,98] also made much effort to perform a calibration in the minimal sensing case of a single camera and IMU. Ref. [99] introduced the open-source Kalibr toolbox for the spatial and temporal calibration of multiple sensors (cameras/IMUs) for the offline estimation of extrinsic parameters within a maximum likelihood estimation framework. However, due to the narrow field of view of the monocular camera, the calibration results will be limited, so Ref. [100] proposed adding multiple additional cameras to assist the calibration based on the monocular IMU-camera system, and combining a multi-camera visual-inertial state estimation algorithm (denoted as Mu-CI) was applied to the sensor platform to perform the calibration, achieving excellent calibration performance. The joint calibration framework of the IMU-camera, as a novel vision-based calibration technique, utilizes the information collected from the frame sequence to estimate the calibration parameters, but the complexity and constraints still pose great challenges to this scheme. On the other hand, light detection and ranging (LiDAR), as an optical measurement instrument, can be used to obtain the surface of an object as a highly redundant discrete point in a three-dimensional coordinate system, and it can also be used to obtain high-precision position information for use in an error calibration of the IMU. Ref. [101] proposed a multi-feature based method that combines the advantages of point/sphere, line/cylinder and plane features in LiDAR scan data for the field calibration of LiDAR-IMU systems without using any artificial targets or specific facilities. First, the calibration of LiDAR extrinsic parameters is performed by estimating geometric features and solving a multi-feature geometric constraint optimization problem. Then, the relationship between the LiDAR and the IMU intrinsic calibration parameters is determined through a coordinate transformation. Finally, the full information maximum likelihood estimation method is applied to solve the optimization problem of IMU intrinsic parameter calibration. Although their calibration performance is good, they usually have some disadvantages, such as the problem of field real-time calibration of the overall system, and the newly developed 3D-LiDAR will lead to a very complex transformation model between LiDAR and IMU [102].

In addition, many researchers have also introduced deep learning techniques into the calibration of inertial sensors; Ref. [103] proposed using machine learning techniques to regress the velocity vector with linear acceleration and angular velocity as input. Ref. [104] proposed to model the IMU calibration as a Markov Decision Process and use reinforcement learning to implement the regression of the calibration parameters. Ref. [105] proposed a lightweight and efficient deep convolutional neural network Calib-Net for low-cost IMU calibration. Calib-Net employs dilated convolutions for spatiotemporal feature extraction, learning to generate gyroscope measurement compensation dynamically, and introducing a mathematical calibration model to design the training and calibration framework. The introduction of deep learning can reduce human intervention and help achieve autonomous systems. Since deep learning methods are data driven, their internal algorithms are more difficult to interpret from a physical and geometric perspective than traditional methods such as observability analysis, which is a natural difference between deep learning and geometric model-based methods [106]. In addition, the generalization ability of the network to different types of IMU proposed by some relevant studies is still limited and the real-time performance is poor. As mentioned above, we briefly analyzed the current situation of calibration technology, introduced some representative cutting-edge calibration technologies that can reflect future development, and defined the brief contents and calibration characteristics of various methods.

## 5. Conclusions

MEMS inertial sensors have a series of advantages such as a low cost, small volume, light weight, low power consumption, mass production, strong impact resistance, high reliability and relatively long service life. Their appearance and continuous development have extensively promoted the further application and development of carrier attitude measurement [107,108,109]. AHRS based on the MEMS inertial sensor/magnetic sensor combination has attracted more and more attention because of its significant advantages, i.e., being low cost and convenient, and the reliable provision of carrier attitude information. This paper gives a brief overview of different MEMS inertial sensor calibration techniques, from traditional calibration schemes to sensor fusion, and covers the latest advances in calibration techniques, but still cannot provide a comprehensive introduction and analysis of related technologies. MEMS inertial sensors are often affected by various factors, sometimes with significantly nonlinear characteristics. Furthermore, the interaction of environmental factors and dynamic changes will have different effects on the sensor. It is foreseeable that the calibration of inertial sensors in complex environments is characterized by nonlinearity and dynamics, which is very different from traditional calibration. While some research has been produced in the field of MEMS inertial sensor calibration, a complete solution suite for most problems still does not exist, and there is still a lot of room for improving calibration schemes. We also summarize the following points to better promote the prospect of calibration technology in MEMS inertial sensors:

- The selection or improvement of appropriate calibration methods will depend on the accuracy requirements and application scenarios;
- The combination of common calibration methods or sensor fusion can improve the performance.

To sum up, the error cannot be completely eliminated under the current technical conditions. All we can do is to make the measurement results closer to the actual value through calibration technology. In the future, determining how to calibrate MEMS inertial sensors in a complex environment is still an important development direction of MEMS inertial sensor calibration technology.

## Figures and Tables

**Table 1:** Approximate range of key parameters of gyroscopes for different classes of use.

Performance Indicators	Strategic Level	Navigation Level	Tactical Level	Commercial Level
Scale Factor Stability (Ppm)	<1	1~100	100~1000	>1000
Zero-Bias Stability (°/h)	<0.005	0.01~0.15	0.15~15	>15
Random Walk (°/$\sqrt{h}$)	<0.01	0.01~0.05	0.05~0.5	>0.5
Rate Noise Density (°/s/$\sqrt{\mathrm{Hz}}$)	<0.001	0.001~0.005	0.005~0.01	>0.01
Range (°/s)	>500	>500	>400	50~1000

**Table 2:** Approximate range of key accelerometer parameters for different classes of use.

Performance Indicators	Strategic Level	Navigation Level	Industrial Level	Consumer Level
Noise (mg/$\sqrt{\mathrm{Hz}}$)	<0.1	<0.7	<5	5
Power Consumption (mA)	<25	<1	<12	1
g-Range (g)	±20	±200	±200	±18
Bandwidth (kHz)	0.33	22	3.2	1

**Table 3:** Approximately optimal nine attitudes for calibrating accelerometers [76].

Posture No.	Description	Illustration
1	*x*-upward	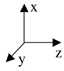
2	*x*-downward	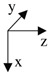
3	*y*-upward	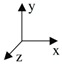
4	*y*-downward	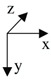
5	*z*-upward	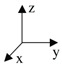
6	*z*-downward	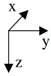
7	*x*-east, *y*-north-upward with $45^{\circ }$ pitch, *z*-south-upward with $45^{\circ }$ pitch	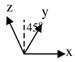
8	*y*-east, *z*-north-upward with $45^{\circ }$ pitch, *x*-south-upward with $45^{\circ }$ pitch	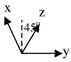
9	*z*-east, *x*-north-upward with $45^{\circ }$ pitch, *y*-south-upward with $45^{\circ }$ pitch	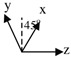

**Table 4:** Comprehensive overview of inertial sensor calibration methods. (Note: Acc—accelerometer; Gyro—gyroscope; Mag—magnetometer).

Group	Type—Reference	Advantage	Disadvantage	Remark
Nonautonomous Calibration	Acc and Gyro—[84]	There is a pre-estimation of the initial value, using the turntable	26-position calibration, cumbersome operation, and unusable when the triplet is seriously dislocated	Increased rotational excitation via a single-axis turntable
	Acc and Gyro—[86]	Combined calibration, taking triplet errors into account	Accuracy needs to be improved	Based on dot product invariant method
	Acc and Mag—[69]	Reduced misalignment errors through the use of turntable and precision aluminum cubes	Cannot be used when the inclination is close to $\pm 90^{\circ }$	Cumbersome operation—need to acquire data of 12 positions and 36 axes
	Acc—[71]	Only bias is dynamically calibrated to simplify the dynamic model	Leverage arm effect is not considered	Bias online calibration based on time-varying Kalman filter
	Acc and Gyro—[76]	Optimal calibration rotation scheme, considering triple calibration deviation	cumbersome, involuntary	By fixing the axial rotation, the triplet bias calibration is resolved
	Acc and Gyro—[87]	Taking into account scale factors and deviations at different temperatures	Complicated operation, inconvenient for engineering application	Real-time thermal calibration by using rate table, thermal chamber and cube housing
	Acc and Gyro—[72]	Take into account the accelerometer lever arm effect (add two variables for the accelerometer)	The operation is complex and time-consuming, and the amount of calculation is large	Two-axis turntable and requires continuous rotational excitation
	Gyro—[85]	Taking into account scale factors and deviations at different temperatures	Complicated operation, inconvenient for engineering application	Real-time thermal calibration by using rate table, thermal chamber and cube housing
	Acc and Gyro—[75]	Taking into account the lever arm effect	Turntable and angular acceleration estimator, limited usage scenarios	Propose TUKF to estimate model parameters
Autonomous Calibration	Acc—[77]	The operation is simple, and the density function does not need to know too much	The calculation amount is large in the nonstationary state	The misalignment error is not considered
	Acc and Gyro—[79]	Minimize model parameters using Newton’s method	The actual calibration of the gyroscope is not described, the initial value needs to be determined	Accelerometer calibration is based on a cost function
	Acc—[68]	Tilt angle information is not required	Only six calibration parameters are estimated, which is cumbersome to operate	Using a shell and three-way milling vice, with reduced initial value requirements
	Gyro—[93]	Creating virtual rate on the gyroscope by using its drive electrodes	Calibration method requires gain adjustment in the output and is affected by aging	Using the condition that the phase shift of the vibration mode is proportional to the excitation rotation rate
	Acc and Gyro—[90]	Taking into account the triplet error between the gyroscope and the accelerometer	Gyroscope accuracy depends on accelerometer calibration accuracy	Using gravity vector calculated from gyroscope output to calibrate
	Acc—[61]	Introduce white Gaussian noise, assuming a Gaussian process	Offline calibration, requires knowledge of the random variable density function	Accelerometer offline calibration based on maximum likelihood estimation
	Gyro and Mag—[91]	Utilizing magnetic field vectors as low cost gyro references	High requirements for magnetic field stability	With sufficient rotational excitation, the magnetic field can be used as a calibration reference
	Acc—[65]	Accurate parameter estimation within three iterations, with low requirements for initial values	Lacks nonlinear corrections and only assumes positive scale factors	Can be implemented on wearable devices with limited computing power
	Acc and Gyro and Mag—[66]	Free rotation plane, pre-estimated initial value	Numerical integration may introduce errors	Known rotations in multi-position calibration are utilized
	Acc and Gyro—[58]	Static parameters are used as initial values and judged	Must be rotated in a fixed configuration	Adopt dynamic and static combination
	Acc—[64]	There is no need to explicitly derive the error model and estimate the error parameters	A large amount of data needs to be trained in the early stage	A new calibration algorithm based on neural networks
	Acc—[80]	Using GNLS method, the convergence speed is fast	30 positions, tedious operation	The accuracy is slightly higher than using LM and GN

## Data Availability

Not applicable.
